# Retraction: A hierarchical Ca/TiO_2_/NH_2_-MIL-125 nanocomposite photocatalyst for solar visible light induced photodegradation of organic dye pollutants in water

**DOI:** 10.1039/d5ra90141c

**Published:** 2025-11-25

**Authors:** Najmeh Ahmadpour, Mohammad Hossein Sayadi, Shahin Homaeigohar

**Affiliations:** a Department of Environmental Engineering, Faculty of Natural Resources and Environment, University of Birjand Birjand Iran mh_sayadi@birjand.ac.ir +98 5632254066 +98 5632254068; b School of Science & Engineering, University of Dundee Dundee DD1 4HN UK; c Nanochemistry and Nanoengineering, Department of Chemistry and Materials Science, School of Chemical Engineering, Aalto University Kemistintie 1 00076 Aalto Finland

## Abstract

Retraction of ‘A hierarchical Ca/TiO_2_/NH_2_-MIL-125 nanocomposite photocatalyst for solar visible light induced photodegradation of organic dye pollutants in water’ by Najmeh Ahmadpour *et al.*, *RSC Adv.*, 2020, **10**, 29808–29820, https://doi.org/10.1039/D0RA05192F.

The Royal Society of Chemistry hereby wholly retracts this *RSC Advances* article after being contacted by the University of Dundee regarding an investigation into the reliability of the data presented in this *RSC Advances* article.

Concerns had been raised with the integrity of XRD data in Fig. 1a and 7c, the FTIR spectra in Fig. 1b, and the TOC analysis in Fig. 8.

The University of Dundee obtained the raw data for Fig. 1a, b, and 7c, but they were not able to obtain the raw data for Fig. 8.

Their investigation concluded that in Fig. 1a, the same data was used to produce multiple XRD traces. They concluded that in Fig. 1b, the Ca data appears to be identical over a substantial range of wavelengths, that the same data was reused in all plots for wavelength beyond ≈2000 cm^−1^, and that the noise appears to be identical. They found that in Fig. 7c, independent measurements were performed only for ≈25° < 2*θ* < ≈26°, ≈31° < 2*θ* < ≈33°, and ≈34° < 2*θ* < ≈35°, and potentially for ≈57° < 2*θ* < ≈58°. The remaining spectra seem to have been copied in several areas with some rescaling.

We requested the raw data from the authors, but they have not provided it, and claim this data was provided by a third party.

Given the significance of these concerns, the Editor has lost confidence that the findings presented in this paper are reliable.

The authors were informed about the retraction of the article. Mohammad Hossein Sayadi has not agreed with the decision, the other authors have not responded.

Signed: Laura Fisher, Executive Editor, *RSC Advances*

Date: 14th November 2025

